# Fabrication of Breathable Coating and Its Hydrophobization Applied for the Rust Stabilization of Weathering Steels

**DOI:** 10.3390/polym18111379

**Published:** 2026-06-02

**Authors:** Junyi Gao, Weichen Xu, Binbin Zhang, Donald Terry Greenfield, Rongling Zhang, Baorong Hou

**Affiliations:** 1 State Key Laboratory of Advanced Marine Materials, Shandong Key Laboratory of Marine Environmental Corrosion and Bio-Fouling, Institute of Oceanology, Chinese Academy of Sciences, Qingdao 266071, China; 2 School of Materials Science and Engineering, Qilu University of Technology, Jinan 250353, China; 3 National and Provincial Joint Engineering Laboratory of Road & Bridge Disaster Prevention and Control, Lanzhou Jiaotong University, 88 An’ning West Road, Lanzhou 730070, China

**Keywords:** weathering steel, rust layer, rust stabilization, breathable coating, SiO_2_ nanoparticle, hydrophobization

## Abstract

The self-formed rust layer is significant for weathering steels because their corrosion resistance in a marine atmospheric environment mainly relies on the stability, uniformity and compactness of the rust layer. However, the initial stage of rust formation is vulnerable and prone to being disturbed by the external environment, compromising the protectiveness of the rust layer at a later stage. Therefore, weathering steel often requires the application of rust stabilization techniques. This study has developed a waterborne polyurethane (WPU)-based coating incorporated with mesoporous/hollow SiO_2_ nanoparticles, acting as the primary components for the construction of pathways for gaseous H_2_O and O_2_, as well as for Cl^−^ dissolved in moisture, while blocking liquid water. Salt spray was applied to accelerate the rust formation process, and rust can form beneath the coating, which provides shelter for rust formation against the external environment. Hexamethyldisilazane (HMDS) was applied to further hydrophobize the nanoparticles, and a hydrophobic surface with self-cleaning properties was achieved. The hydrophobized and non-hydrophobized coatings with different thicknesses (10–80 µm) were systematically compared: the morphology of the rust layer and coating surface after salt spray was investigated, the ability of the rust layer to inhibit chloride ingress was compared, and the electrochemical behaviors were analyzed. This study presents a new strategy for weathering steel rust stabilization that features maneuverability, environmental friendliness and low cost.

## 1. Introduction

Corrosion of infrastructure has attracted public attention globally [1,2]. Weathering steel is a significant material and is widely applied in infrastructure (industries and constructions), which possesses good corrosion resistance via a dense and stable rust layer [3,4,5]; therefore, it can be used in an atmospheric environment in an uncoated (unpainted) state. However, the development of naturally formed protective rust layer normally requires an extended period (often in several years [6,7]), and the environmental factors such as air pollution, rainwater and its local accumulation, combined with structural complexities, can impede the development of a protective rust layer [7,8,9,10,11].

Rust stabilization technologies are necessarily required to promote the protectiveness of the rust layer or accelerate its formation process. The primary available technologies involve spray-based and coating-based methods. Spray-based techniques typically involve spraying an aqueous solution containing soluble salts onto the steel surface [12,13,14]. The key elements (e.g., CuSO_4_, FeSO_4_, Na_2_WO_4_, Na_2_MoO_4_) in the salts may accelerate the formation of rust and promote the conversion of preliminary corrosion products into the stable α-FeOOH [15,16]. However, it needs to be addressed that the presence of typical heavy metal ions such as Cr^3+^ would induce environmental concerns [13,15]. In addition, periodic spraying of water can also promote the formation of a stable rust layer by increasing the frequency and number of wet–dry cycles, which is easy to operate with low cost. However, its effectiveness is influenced by local environmental conditions, spraying cycles and the rust layer evolution process, and the rust layer formed on different surface conditions such as welds and cut edges tends to be less effective [13,17,18,19].

Coating technology accelerates the formation of a protective rust layer by incorporating rust-promoting agents (e.g., Cr_2_(SO_4_)_3_ and CuSO_4_) [6]. Permeable coating is also applied, which allows a controlled amount of corrosive media to penetrate, promoting rust formation while preventing the dripping and splashing of rust runoff [20,21]. The coating process can be complex, and the coating film may fail prematurely as a result of water penetration [21,22].

There are also other rust stabilization technologies reported in the literature, including synthesizing SiO_2_ on the rust layer to achieve hydrophobicity [23] and the development of microalloying steel (e.g., Ce, Ca, V) to promote the formation of a dense and protective rust layer [24,25,26]. Other attempts include cleaning the rust layer surface of weathering steel using continuous-wave laser or abrasive water jet to improve the corrosion resistance of the rust layer [27].

In terms of the base material for coating, polyurethane (PU) is one of the most widely used high-performance organic coating materials. It offers wear resistance, high elasticity, excellent thermal stability, mechanical performance and resistance to corrosion. These properties make it widely applied in marine engineering, the oil and gas industry, etc. [28,29]. However, traditional solvent-based PU coatings release a large amount of VOCs during production and application. Waterborne polyurethane (WPU) uses water instead of organic solvents, so the VOC emissions are effectively decreased. It is also non-toxic, non-flammable and easy to apply—fitting well with the green manufacturing demands [30,31]. Meanwhile, with fossil resources running out and the push for sustainable development, bio-based waterborne polyurethane has become a hot research topic [32]. Bio-based polyols made from renewable resources like vanillin, castor oil or algae can partly or fully replace petroleum-based polyols in PU production [32,33]. WPU has advantages in terms of safety, reliability, environmental degradability and cost. Table 1 summarizes different types of polyurethane-based coatings and their properties.

Recently, the authors of this work reported a novel technique based on the “breathable coating” inspired by its application in the textile industry, which selectively allowed water vapor but not liquid water to penetrate through and provided a shelter for the formation of a rust layer beneath the coating film [44]. It improved the quality of the rust layer, but the hydrophilicity of the WPU substrate led to early blistering and compromised its durability. Hydrophobicity can usually be achieved by increasing surface roughness and reducing surface energy, minimizing the contact area between water and the substrate and inhibiting the adhesion, retention, penetration and diffusion of liquid water [45,46]. Meanwhile, water droplets that roll off in a spherical shape can adsorb and carry away dust and contaminants attached to the surface, thereby keeping the coating clean [47]. Therefore, the hydrophobization of the coating would be an effective and promising way to enhance its performance.

Many studies have utilized various hydrophobically modified micro-/nanoscale particles (e.g., SiC, TiO_2_, PTFE) as fillers to obtain hydrophobic coatings [48,49,50]. This work involved the synthesis of hollow silica nanoparticles (HSNs) followed by hydrophobic modification. Both modified and unmodified HSNs were used as fillers and blended with WPU to form breathable coatings. The permeability (breathability) of the coatings and the protectiveness of the rust initially formed beneath the coating were analyzed. The hydrophobically modified HSNs facilitated the formation of a hydrophobic and breathable coating, which demonstrated an enhanced barrier effect against the ingress of liquid water, offering shelter during the process of rust layer formation beneath the coating film. This research improves the performance of the breathable coating, especially in terms of waterproof properties, and provides a reference for the development of new rust stabilization technology on weathering steels.

## 2. Materials and Methods

### 2.1. Materials

The material used in this work was Q420qDNH weathering steel (see Table 2 for its chemical composition). The sample dimensions were 50 mm in length, 25 mm in width and 3 mm in thickness. The sample surfaces were successively ground with 600-grit and 1000-grit sandpaper, cleaned with anhydrous ethanol and dried with cold air.

### 2.2. The Fabrication of Hollow SiO_2_ Nanoparticles

At room temperature, a 200 mL ethanol/water mixture with a volume ratio of 0.6 was stirred using a magnetic stirrer, and then 0.40 g of cetyltrimethylammonium bromide (CTAB), used as the templating agent, was added into the solution. After complete and uniform dissolution, 2.50 mL of ammonia solution (providing an alkaline environment) and 2.25 mL of the silica source tetraethyl orthosilicate (TEOS) were added. Under constant stirring conditions, the mixture was left to react for 6 h. Afterwards, the solution was centrifuged, and the collected solid material (presented as white powder) was rinsed with ethanol. It was eventually dried in a vacuum so that hollow SiO_2_ nanoparticles were obtained.

The hollow SiO_2_ particles (2.0 g) were added to 40 mL of absolute ethanol, and the suspension was stirred vigorously until a uniform mixture was obtained. Subsequently, 1.5 mL of the surface hydrophobic modifier hexamethyldisilazane (HMDS) was added, and the mixture was allowed to react under continuous stirring for 6 h. Surface-hydrophobized hollow SiO_2_ nanoparticles were thus produced following centrifugation, washing and drying under vacuum conditions.

### 2.3. The Characterization of Hollow SiO_2_ Nanoparticles

The hollow SiO_2_ nanoparticles, as stated above, were dispersed in ethanol, and the suspension liquid was dripped onto an aluminum foil. After the ethanol evaporated, the nanoparticles on the aluminum foil were sputter-coated with gold, and the morphology was observed using a scanning electron microscope (Hitachi Regulus 8100, Hitachi High-Tech Corporation, Tokyo, Japan) in InLens signal mode with an accelerating voltage (EHT) of 1.00 kV. The suspension liquid of the nanoparticles was also dropped onto copper grids. Once the ethanol evaporated, a JEM-F200 transmission electron microscope (JEOL Ltd., Tokyo, Japan) was applied for the investigation of the structure of the nanoparticles in transmission mode at an accelerating voltage of 200 kV. A Fourier Transform Infrared Spectroscopy (FTIR, Nicolet iS20, Thermo Fisher Scientific, Waltham, MA, USA) was used to record the IR spectrum of the hydrophobized hollow/mesoporous SiO_2_ nanoparticles using the transmission mode (KBr pellet method). The spectrum was collected in the wavenumber range of 400–4000 cm^−1^ at a resolution of 4.00 cm^−1^ with 32 scans.

### 2.4. The Fabrication of the Coating

A total of 2.0 g of SiO_2_ nanoparticles was dispersed in 20.0 mL of anhydrous ethanol, and 2.0 g of waterborne polyurethane (WPU) with a solid content of 40% was added to obtain the SiO_2_/WPU mixture. The waterborne polyurethane (WPU) used in this study was an anionic polyester-based Macklin waterborne polyurethane produced by Shandong Keyuan Biochemical Co., Ltd. (Heze, China), with a solid content of 40%. The coatings were then applied to the steel sample surface using a spray gun at a pressure of 0.2 MPa from a distance of 10–15 cm from the surface. The wet coating required 6 h to cure.

### 2.5. Water Vapor Transmission Assessment of the Coating Film

A cup for the water vapor transmission test was filled with 30 mL of deionized water. The coating film described in Section 2.4 was deposited onto a piece of non-woven fabric and put into the cup. It was subsequently sealed using hot liquid paraffin. Once the paraffin was solidified, the initial mass of the cup was measured and designated as m_1_. The cup was placed in a constant temperature and humidity chamber (TB-80Z, Guangdong Tongbiao Precision Instrument Co., Ltd., Dongguan, China) under conditions of 38 ± 0.5 °C and 90 ± 2% RH for 24 h. Afterward, the mass of the cup was recorded as m_2_.

Equation (1) was employed to calculate the water vapor transmission of the coating film. The symbol P stands for the sample’s water vapor transmission rate (WVTR), with units of mg/(10 cm^2^·24 h). “m_1_” and “m_2_” are expressed in mg. “A” stands for the cup’s effective area for water vapor transmission (28.3 cm^2^).

The series resistance model (Equation (2)) was applied to subtract the water vapor transmission contribution of the non-woven fabric, thereby obtaining the intrinsic water vapor transmission of the SiO_2_/WPU coating alone [51]. “P_coating_” represents the water vapor transmission rate (WVTR) of the coating, and “P_fabric_” corresponds to the WVTR of the fabric.

(1) $$P=\frac{m_{1}\;-{\;m}_{2}}{A},$$

(2) $$P_{\mathrm{coating}}=\frac{1}{\frac{1}{P}-\frac{1}{P_{\mathrm{fabric}}}},$$

### 2.6. Water Contact Angle Measurement

The hollow SiO_2_ particles were placed on a glass slide. A dyed water droplet was then placed on the particles to investigate the hydrophilic or hydrophobic nature of the particles. The water contact angles of the coating surfaces (Section 2.4) were measured using a goniometer (OCA25, DataPhysics Instruments GmbH, Filderstadt, Germany). For each sample, five different locations were selected, and the contact angle was measured at each location. The average of the five measurements was calculated, and their standard deviation was reported.

Common liquid contaminants such as stained water, cola, juice and milk were separately dropped onto the coating surface, and their wetting behavior was observed and photographed. On the coating surface tilted at an angle of 10°, sand was used to simulate the contaminants, while stained water was continuously dripped onto the surface. The self-cleaning capability of the coating was then evaluated by rinsing sand with water.

### 2.7. Salt Spray Test

Coated steel samples with varying thicknesses prepared using the two types of hollow SiO_2_ nanoparticles (hydrophobized and non-hydrophobized), along with uncoated bare steel samples, were placed in a salt spray test chamber (CYP-90, Suga Test Instruments Co., Ltd., Tokyo, Japan), and the test lasted for 28 days. The salt spray test applied a temperature of 35 ± 2 °C (GB/T 10125-2021 [52]), a relative humidity of 98%, and a spray pressure of 0.1–0.15 MPa. A 5.0% NaCl solution was prepared using analytical-grade sodium chloride reagent from Sinopharm Chemical Reagent Co., Ltd. (Shanghai, China), which was used to obtain a neutral salt spray environment. All samples were placed at a 30° tilt in the test chamber.

### 2.8. Characterization of the Rust Layer

Rusted samples after a certain period of salt spray test were sputter-coated with gold and observed using SEM (Hitachi Regulus 8100, Hitachi High-Tech Corporation, Tokyo, Japan) in SE2 signal mode with an accelerating voltage (EHT) of 3.00 kV to examine the surface morphology of the rust layer. The samples were embedded in epoxy resin and cut to reveal the cross-section of the rust layer and then polished using abrasive papers of 600–3000 grit. Subsequently, a scanning electron microscope (SEM) equipped with an energy-dispersive X-ray spectroscopy (EDS, 550i, IXRF Systems, Inc., Austin, TX, USA) was used. It was operated in SE2 signal mode at an accelerating voltage (EHT) of 15.00 kV. The cross-sectional morphology was examined, and the distribution of Fe, O, and Cl within the rust layer was analyzed via mapping mode. The rust formed on the surface of each sample was scraped off and ground into powder. Then, an X-ray diffractometer (XRD, SmartLab, Rigaku Corporation, Tokyo, Japan) was used for phase identification and semi-quantitative analysis. The diffractometer used a copper target, with a diffraction angle scanning range from 10° to 75°, a step size of 0.02°, and a scanning speed of 1°/min.

After a 28-day salt spray test, the open-circuit potential, electrochemical impedance spectroscopy and the potentiodynamic polarization test were carried out on the rust layer using a three-electrode setup. An Ivium CompactStat.h workstation (Ivium Technologies B.V., Eindhoven, The Netherlands) was used, where the working electrode (WE) consisted of a 10 × 10 mm^2^ exposed area, the counter electrode (CE) was a 20 × 20 mm^2^ platinum sheet, and the reference electrode (RE) was a saturated calomel electrode (SCE). The electrolyte was a 3.5 wt% NaCl solution. Once the open-circuit potential (OCP) had stabilized (ΔOCP < 3 mV/5 min) during free immersion, electrochemical impedance spectroscopy (EIS) was carried out. The frequency range was from 10^5^ Hz to 10^−2^ Hz with a 10 mV amplitude. Afterwards, a potentiodynamic polarization test was performed, scanning from −0.25 V to +0.25 V versus the open-circuit potential at a scan rate of 1 mV/s, to obtain the polarization curve of the rust layer. Fitting of the EIS data was performed using ZView software (version 2).

## 3. Results

### 3.1. The Hollow SiO_2_ Nanoparticles and Their Hydrophobization

**Figure 1:**
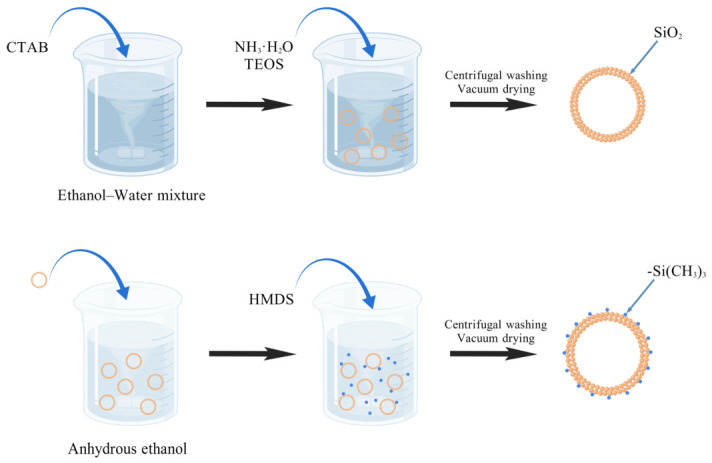
A schematic diagram of the hollow SiO_2_ nanoparticles fabrication process.

The synthesis and hydrophobization method for the hollow SiO_2_ nanoparticles is schematically shown in Figure 1. Section 2.2 already provides the details of the preparation method of the nanoparticle. The micelles formed by the soft template CTAB in the mixed solution of ethanol and water possess hydrophobic tails in the interior and hydrophilic heads in the exterior. Consequently, the interior of the micelles constitutes an ethanol-rich phase, while the exterior is a water-rich phase. The SiO_2_ precursor exhibits higher solubility in ethanol than in water; therefore, TEOS tends to accumulate within the ethanol-rich phase inside the micelles due to similar polarity. The alkaline ammonia facilitates the hydrolysis and condensation reactions of TEOS. The resulting primary SiO_2_ nanoparticles densely pack on the inner surface of the micelles, forming a SiO_2_ shell with a hollow structure.

The molecular structure of hexamethyldisilazane (HMDS) features reactive Si-NH-Si bonds, enabling a chemical reaction with the Si-OH (silanol) groups on the surface of the hollow SiO_2_ nanoparticles as described above, thereby achieving hydrophobic modification of the particle surface. As shown in Equation (3), in an anhydrous ethanol solution, the Si-N bond in HMDS cleaves and undergoes a deamination reaction (releasing NH_3_) with the hydroxyl groups from the Si-OH on the SiO_2_ surface. This process (known as a silanization reaction) generates stable Si-O-Si covalent bonds and grafts the trimethylsilyl groups (-Si(CH_3_)_3_) of the HMDS molecule onto the SiO_2_ particle surface.

(3) $$2\equiv \mathrm{SiOH}+{\left({\mathrm{CH}}_{3}\right)}_{3}\text{-}\mathrm{Si}\text{-}\mathrm{NH}\text{-}\mathrm{Si}\text{-}{\left({\mathrm{CH}}_{3}\right)}_{3}\;\to \;2\equiv \mathrm{Si}\text{-}O\text{-}\mathrm{Si}\text{-}{\left({\mathrm{CH}}_{3}\right)}_{3}+{\mathrm{NH}}_{3}$$

By grafting HMDS onto the surface, the original hydrophilic Si-OH groups of hollow SiO_2_ nanoparticles are replaced with nonpolar -Si(CH_3_)_3_ groups. This substitution greatly lowers the surface energy, making the particle surface hydrophobic.

**Figure 2:**
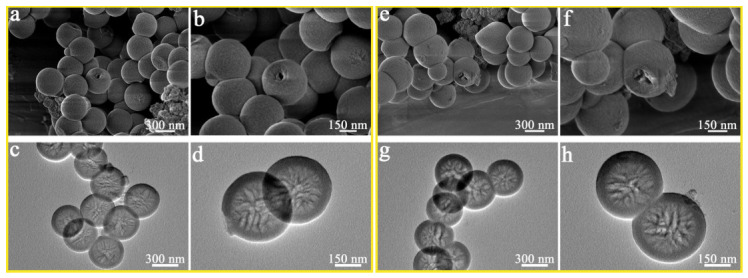
The SEM and TEM images of hollow SiO_2_ nanoparticles (**a**–**d**) and hydrophobic hollow SiO_2_ nanoparticles (**e**–**h**).

Figure 2 presents the SEM and TEM images of the two types of hollow SiO_2_ nanoparticles before and after hydrophobic modification. Both nanoparticles have a well-defined hollow structure with a diameter of ca. 200 nm. The nanoparticle shells are composed of aggregated primary SiO_2_ particles, with numerous inter-particle voids. No significant morphological or structural differences are observed between the two types of nanoparticles, indicating that the hydrophobic modification with HMDS does not alter the hollow structural characteristics.

**Figure 3:**
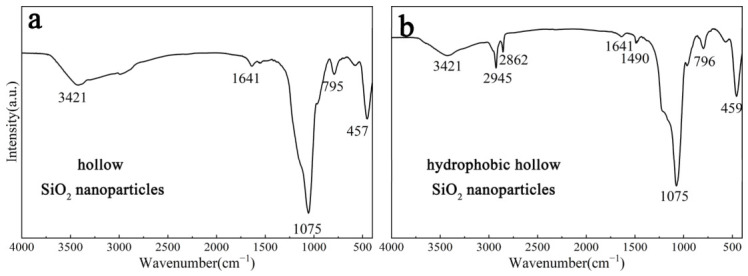
The FT-IR spectrum of the unmodified (**a**) and modified (**b**) hollow SiO_2_ nanoparticles.

The FTIR spectra of the unmodified and modified hollow SiO_2_ nanoparticles are presented in Figure 3. For the unmodified hollow SiO_2_ nanoparticles, the peak at 459 cm^−1^ arises from the bending mode of the Si–O bond. The peak at 796 cm^−1^ arises from the symmetric stretching of the Si–O–Si bond. The peak at 1075 cm^−1^ corresponds to the asymmetric stretching of Si–O–Si linkages [53]. The peak at 1641 cm^−1^ is due to the H–O–H bending vibration of water molecules [53,54]. The broad peak at 3421 cm^−1^ is attributed to the stretching vibration of –OH bonds in adsorbed water [55,56]. For the hydrophobic hollow SiO_2_ nanoparticles, an additional small peak near 1490 cm^−1^ was observed, which is due to the bending vibration of methyl (–CH_3_) groups [57,58]. The peaks at 2862 cm^−1^ and 2945 cm^−1^ are assigned to the symmetric stretching vibration and the asymmetric stretching vibration of –CH_3_, respectively [59]. These features confirm the grafting of hydrophobic methyl groups onto the SiO_2_ surface.

### 3.2. Water Vapor Transmission Rate (WVTR) of the Coating

The specimens (coated and uncoated, non-hydrophobic and hydrophobic) have been numbered accordingly. The specimen codes denote the coating thickness and the type of SiO_2_ nanoparticles used: the W10_Si_, W40_Si_ and W80_Si_ incorporated SiO_2_ hollow nanoparticles as the coating filler with thicknesses of 10, 40 and 80 µm, respectively; the W10_h-Si_, W40_h-Si_ and W80_h-Si_ incorporated hydrophobic hollow SiO_2_ nanoparticles as the filler with thicknesses of 10, 40 and 80 µm, respectively. In all these coatings, the mass ratio of SiO_2_ nanoparticles to WPU was maintained at 1:1. A pure WPU coating designated as W10 served as the blank control.

**Figure 4:**
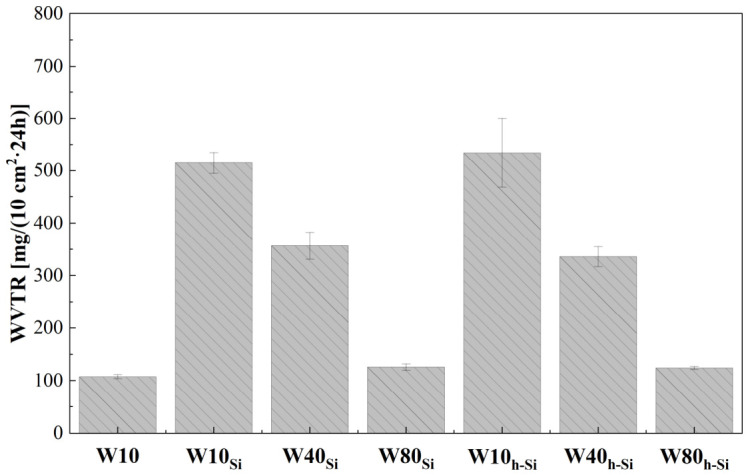
The water vapor transmission rate (WVTR) of different coatings.

Figure 4 compares the water vapor transmission rate (WVTR) of the coating films for all specimens. All the coatings incorporated with hollow SiO_2_ nanoparticles showed higher WVTR than the blank control (W10), and the WVTR decreased with increasing coating thickness. The hydrophobized or non-hydrophobized nanoparticles did not exert a significant influence on the WVTR for coatings with the same thickness.

### 3.3. Water Contact Angle of the Coating

**Figure 5:**
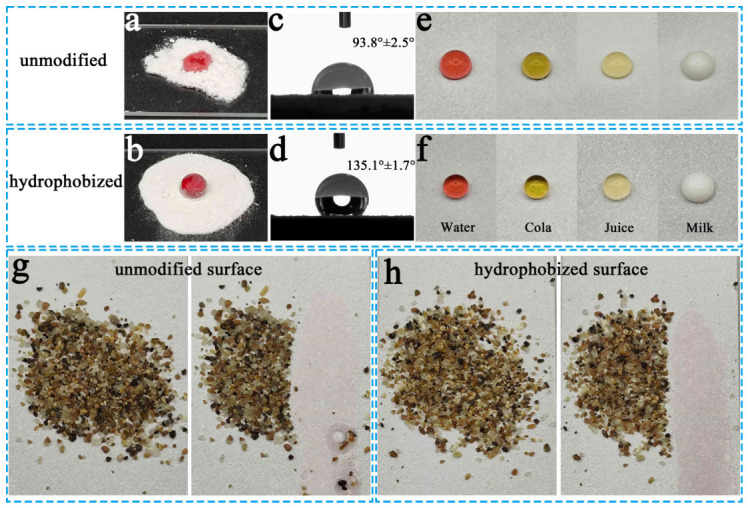
Water droplets on the surfaces of the two types of nanoparticles (**a**,**b**); static water contact angle measurement on coating surfaces prepared from the two types of nanoparticles (**c**,**d**); common liquid droplets on the surfaces of the two coatings (**e**,**f**); self-cleaning capability of the two coatings (**g**,**h**).

Figure 5a,b show the state of stained water droplets on the surfaces of hollow SiO_2_ nanoparticles before and after hydrophobic modification. The unmodified nanoparticles were hydrophilic, whereas the water droplets remained spherical on the powder surface after hydrophobic modification. The static water contact angles on the coating surfaces incorporating the two different nanoparticles are shown in Figure 5c,d. After modification, the coating surface became more hydrophobic, with the contact angle rising from 93.8 ± 2.5° to 135.1 ± 1.7°, and the scale bar was calculated based on five different locations on each surface. The shape of some common liquid droplets on the coating surfaces shown in Figure 5e,f further confirms the improvement of hydrophobicity. The self-cleaning behavior of both coatings was tested with sand particles acting as solid contaminants (Figure 5g,h). Compared with the unmodified coating, solid contaminants are more efficiently removed from the surface of the hydrophobic coating.

### 3.4. Macroscopic Observation After Salt Spray Test

**Figure 6:**
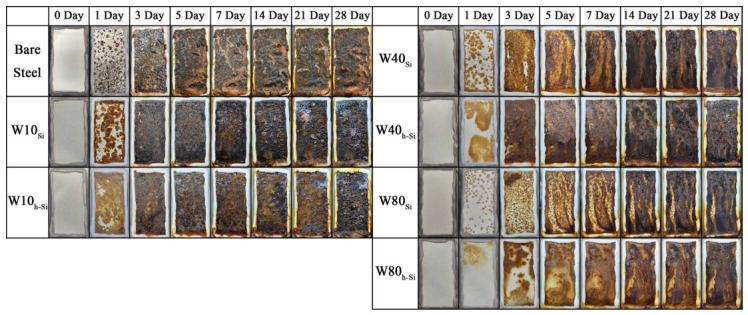
The macroscopic images of the bare surface and coated surface (hydrophobized and non-hydrophobized) with different coating thicknesses during the salt spray test (the size of the sample was 50 × 25 mm^2^).

After different periods of salt spray test, the macroscopic images of the sample surface are provided in Figure 6. The bare metal surface rusted rapidly, with loosely adherent rust layers (flaked and partially detached at day 5 and after). In contrast, the occurrence of rust flaking was markedly reduced for the coated specimens.

During the salt spray test, a cotton bud was periodically used to check whether the rust layer was exposed or not. The W10 group showed that the cotton bud was stained and rust layers were exposed as early as the seventh day. The W40 group began to exhibit local coating damage on the 14th day, while no obvious stain on the cotton bud was observed on the W80 group until the end of the test.

A clear delay in rust development was observed with greater thickness for both coatings. The rust exhibited a speckled morphology for the non-hydrophobized group. The size of the speckle and the coverage of the rust were both decreased with increasing thickness, which is ascribed to the decreasing WVTR and inhibited penetration of corrosive media (chloride ions and oxygen).

On day 14, scar-like defects began to appear on W10_Si_, indicating the localized failure of the coating (as a result of coating damage after rust expansion) and the premature exposure and damage of the rust layer. By day 28, the surfaces of both W10_Si_ and W40_Si_ were completely covered by a rust layer (with scar-like defects presented in the rust layer on W10_Si_, while the rust layer on W40_Si_ seemed to be less dense), whereas W80_Si_ had not yet developed a complete rust layer.

Unlike the isolated speckled morphology observed on the coatings in the non-hydrophobized group, the rust formed on the hydrophobic group during the initial stage exhibited a relatively continuous rust layer, which can favor the subsequent development of a more uniform rust layer. However, for the relatively thin coating (W10_h-Si_), the damage to the coating still occurred by day 14, resulting in irregular scar-like defects on the rust layer. When the coating thickness increased (W40_h-Si_), a more uniform and complete rust layer was formed after 28 days. For the thickest coating (W80_h-Si_), the rust layer formed beneath the coating still remained incomplete after 28 days.

### 3.5. Microscopic Observation of the Coating and Rust Layer

**Figure 7:**
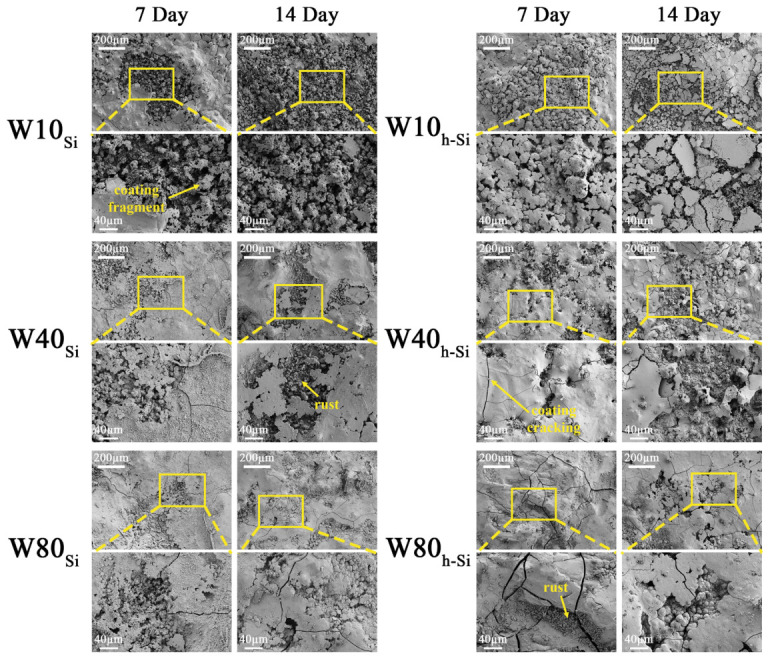
The SEM images of the specimen surface covered by hydrophobized and non-hydrophobized coatings with different thicknesses after the 7-day and 14-day salt spray test (in each box, the image on the right was a magnification of the left).

After 7 and 14 days of salt spray test, the coating surface morphologies investigated by SEM are displayed in Figure 7. Generally, the failure of the coating was initiated with the appearance of cracks, followed by fragmentation and spallation of the coating around the cracks. For the W10_Si_ sample, the coating surface exhibited failure of a large area and the rust was clearly exposed on day 7, and the coating almost fell off completely on day 14. The failure appeared as fragments for the W40_Si_ sample on day 7, while a significant portion of the coating still remained on day 14. Local spallation with cracks was observed on the W80_Si_ sample on day 7, while the failure mode still remained the same on day 14, and the rust was only locally exposed.

For the hydrophobized group, the rust was also largely exposed on day 7 for the W10_h-Si_ sample, and the failure became fragmented on day 14, but the rust layer was still partially covered, indicating a delayed failure of the coating compared to the premature failure of W10_Si_. The failure mode can be described as local spots with a few cracks on day 7 for the W40_h-Si_ sample, while a large area of failure appeared on day 14. The failure mode was in cracks on day 7 for the W80_h-Si_ sample, and the exposed rust was also in small areas. The rust particles seemed to be pushed out through the cracks (day 7) and distributed near these cracks, while there was no rust elsewhere.

**Figure 8:**
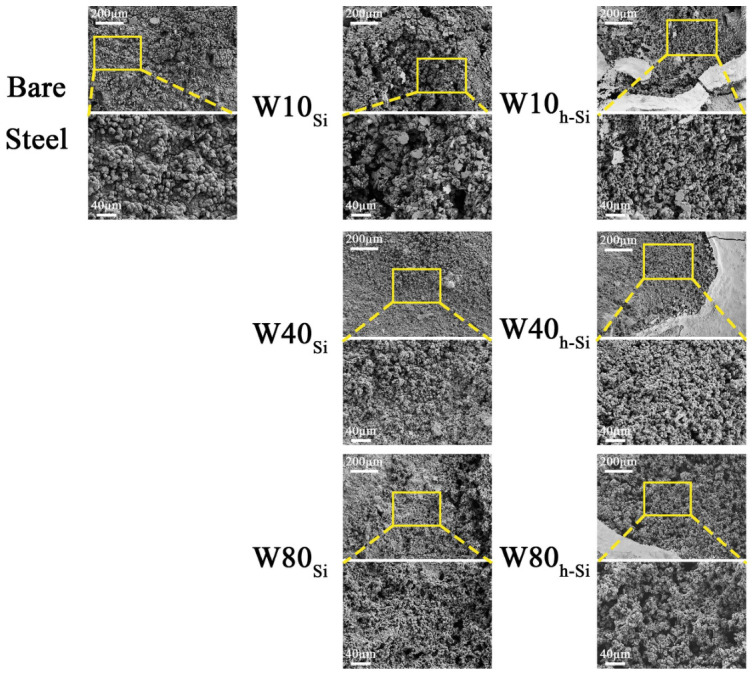
The SEM images of the rust layer formed on the sample surface with hydrophobized and non-hydrophobized coatings (after coating failure) after 21 days of salt spray test.

During the period from day 1 to day 14 of the salt spray test, the rust development was in the initial stage. By day 21, a complete rust layer was formed on the surfaces of most samples, but the degree of uniformity was different. Therefore, the analysis of the rust layer morphology was conducted after day 21. The rust morphologies of the samples after 21 days of salt spray test are shown in Figure 8. The rust particles on the bare steel were relatively large in size and appeared loose, with a large number of voids and cracks. For the W10_Si_, the rust also appeared loose, whereas the structure of the rust on W40_Si_ was obviously denser than that of W10_Si_. The rust on W80_Si_ was not yet fully developed (see Figure 6), and small voids were present. The amount of voids in the rust layer was reduced on W10_h-Si_ than W10_Si_, while it presented as a finer and more uniform structure on W40_h-Si_. In comparison, a loose structure with a large number of voids was presented on W80_h-Si_.

### 3.6. The Compositional Analysis of the Rust Layer

**Figure 9:**
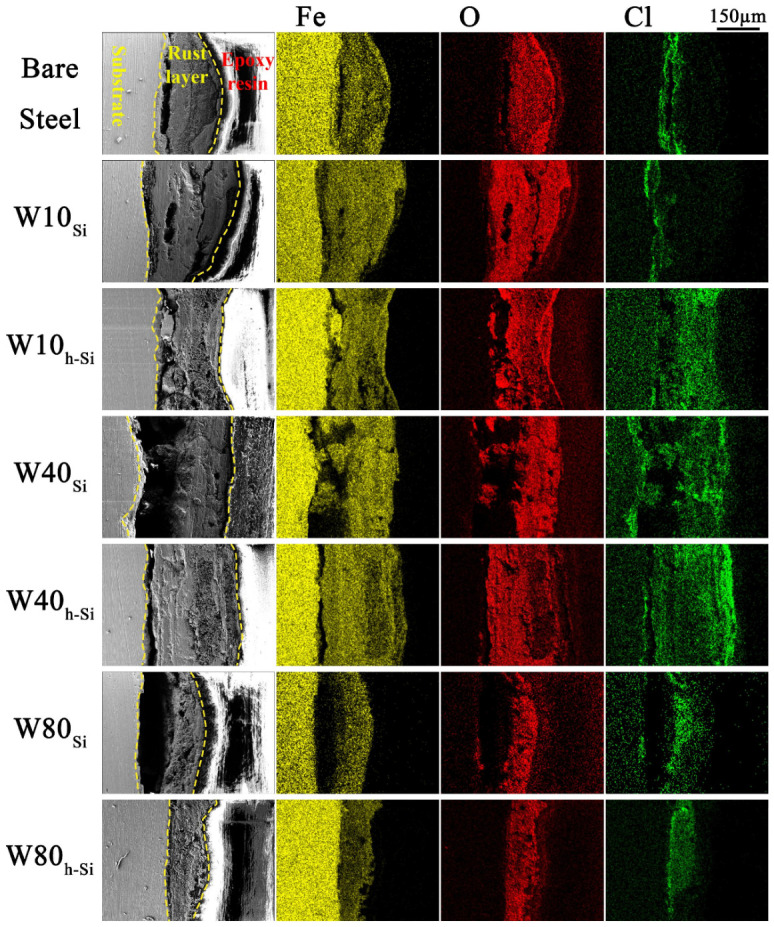
The SEM images of the rust layer cross-sections of different specimens after the 28-day salt spray test with the corresponding EDS mappings of Fe, O and Cl.

Figure 9 presents the cross-sectional views and EDS mappings of the rust layers formed on different specimens after 28 days salt spray test. The oxygen enrichment and iron depletion clearly distinguish the range of the rust layer. The detachment of the rust layer from the steel substrate was caused by grinding.

In the bare steel specimen, chloride was distributed in the inner region of the rust layer, indicating complete chloride ingress through the rust layer, which threatened the substrate, and it was similar for the W10_Si_ specimen. The W40_Si_ specimen presents the chloride distribution through the thickness of the rust layer without clear local accumulation either in the inner or the outer layer, while chloride accumulation in the inner part was observed on the W80_Si_ specimen. The chloride distribution was also all over the rust layer without noticeable local accumulation for W10_h-Si_ and W80_h-Si_ specimens, but it was very clear for the W40_h-Si_ specimen that chloride was largely distributed in the outer layer of the rust and depleted in the inner layer, indicating that chloride ingress was blocked.

**Figure 10:**
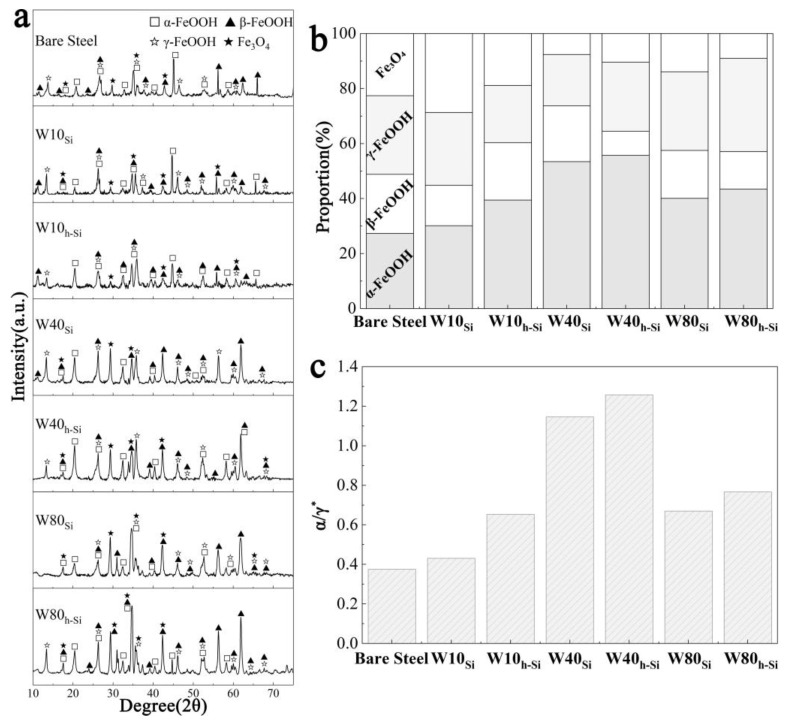
XRD analysis on the rust of different specimens after 28 days salt spray test ((**a**) XRD patterns of the rust layers; (**b**) semi-quantitative XRD analysis; (**c**) mass ratio of α-FeOOH to the sum of γ-FeOOH, β-FeOOH and Fe_3_O_4_ (denoted as α/γ*)).

Additionally, XRD analysis (semi-quantitative analysis) was performed on the rust powders collected from the samples after the 28-day salt spray test. Previous research employed the ratio of the amount of α-FeOOH to the combined amount of γ-FeOOH, β-FeOOH and Fe_3_O_4_ (designated as α/γ*) as a metric for the assessment of the rust layer stability and protective ability on weathering steel [60]. An α/γ* value greater than one indicates good protective performance. All samples exhibited the corrosion products α-FeOOH, γ-FeOOH, β-FeOOH and Fe_3_O_4_ within their rust layers, as shown in Figure 10. The α/γ* values for W40_Si_ and W40_h-Si_ were both greater than one, and the α/γ* value for W40_h-Si_ was the highest at 1.26.

### 3.7. Electrochemical Analysis on the Rust Layer

**Figure 11:**
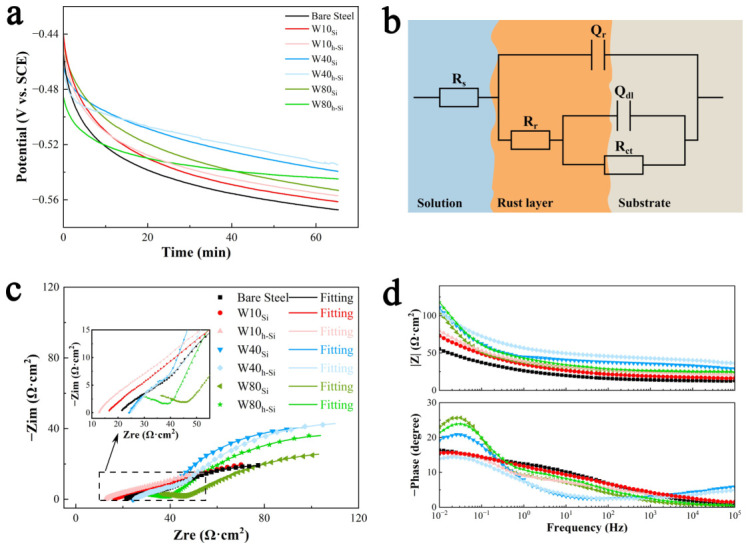
The results of electrochemical measurements on different samples after the 28-day salt spray test: (**a**) the curves of OCP vs. time; (**b**) the equivalent circuit used to fit the EIS results; (**c**) Nyquist plots; (**d**) Bode plots.

The electrochemical data obtained from different samples after a 28-day salt spray test are shown in Figure 11. In a 3.5 wt% NaCl electrolyte, the open-circuit potential of all samples initially decreased and stabilized after approximately 1 h (Figure 11a). Afterwards, electrochemical impedance spectroscopy (EIS) tests were conducted. Figure 11b illustrates the equivalent circuit. R_s_ is the resistance of the solution, R_r_ is the rust layer resistance, and R_ct_ is the charge transfer resistance. A constant phase element (CPE) was employed instead of a pure capacitor. The Nyquist and Bode diagrams are given in Figure 11c,d.

Table 3 presents the fitting results according to the EIS data. Defects such as cracks and pores within the rust layer would reduce its compactness and provide pathways for chloride ions in the solution to penetrate toward the weathering steel surface. A denser rust layer would inhibit the ion transport, leading to a higher R_r_ value. The value of R_r_ serves as a reflection of the inhibition of chloride ingress and rust layer protectiveness. A higher R_ct_ generally corresponds to a lower metal dissolution rate. The W40 group exhibited higher values of stabilized open-circuit potential as well as higher R_ct_ and R_r_ values than all the other groups (bare steel, W10 and W80). Specifically, W40_h-Si_ demonstrated the highest R_ct_ and R_r_ values. In addition, W40_h-Si_ showed the smallest Y_r_ and Y_dl_ values, and its dispersion exponents n_r_ and n_dl_ were the highest, indicating that the rust layer on its surface possessed lower porosity and higher uniformity.

**Figure 12:**
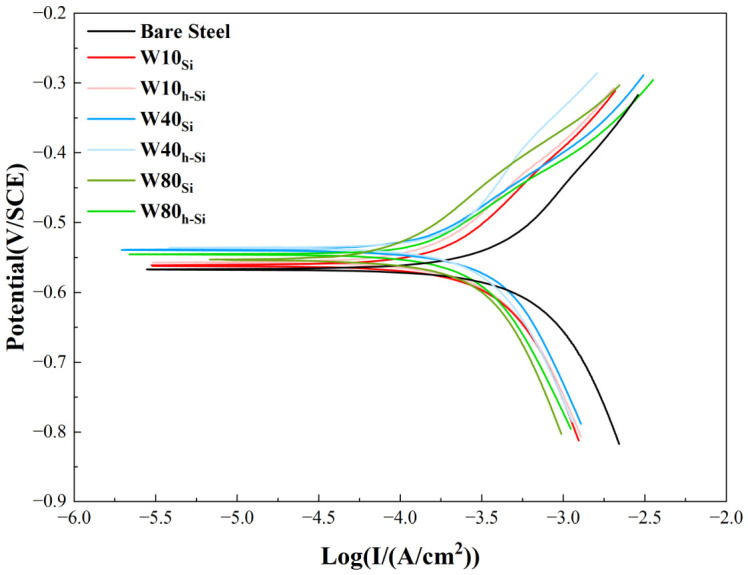
Polarization curves of different samples after 28 days of salt spray test.

Figure 12 presents the electrochemical polarization curves of the different samples after 28 days of salt spray test. Table 4 lists the E_corr_ and I_corr_ values calculated from the polarization curves. All the coated samples (W10, W40 and W80) presented higher E_corr_ values than the bare steel. The W40 group showed obviously lower corrosion current density than the other samples, implying good protectiveness of the rust layer formed beneath the coating film. The hydrophobically modified sample W40_h-Si_ presented the best corrosion resistance, and the corrosion current density was only 17% of that of the bare steel.

## 4. Discussion

### 4.1. Rust Development Beneath Coatings

As a physical barrier, a coating is inherently expected to block corrosive media such as H_2_O, O_2_, and Cl^−^ dissolved in moisture [61]. However, due to the special corrosion-resistant mechanism of weathering steel that relies on the protectiveness of a self-formed rust layer, the complete blocking of the corrosive media would compromise the self-protection ability of weathering steels [62]. The ideal coating designed for the stabilization of self-formed rust layer should possess selective permeability, i.e., allowing the ingress of necessary corrosive media (H_2_O, O_2_, and Cl^−^ dissolved in moisture) that were required to form the rust layer (preferentially in vapor state) while effectively preventing the ingress of undesired liquid water and dust (the main factors to compromise the protectiveness of the rust layer). This is especially significant in the initial stage of the rust formation process, which is vulnerable but crucial for the following rust formation stage. It has already been found that the uniformity of the initial rust layer is very likely to result in a compact and uniform mature rust layer [21,44]. This is the core idea of the designed coating film in this work.

The hollow SiO_2_ nanoparticles in this work can construct nanoscale channels within the coating, which selectively allow the transport of corrosive media in vapor state. The effective connection from the outer surface of the coating all the way to the substrate/coating interface is the key to breathability, while those built halfway cannot act as effective pathways. Additionally, the uniform distribution of nanoparticles in a thick coating is difficult, resulting in the tortuosity of the effective pathways. As shown in Figure 4, water vapor transmission rate (WVTR) is inversely related to the coating thickness, because the longer distance between the outer surface of the coating and the substrate/coating interface would increase the difficulty in constructing the effective pathway for water vapor. It was also demonstrated by the delayed rust development beneath thicker coatings (see Figure 6). Although a thick coating can enhance the physical barrier effect, it significantly compromises the breathability [63,64], which is detrimental to the sustained growth and evolution of the rust layer. However, the excessively fast rust development process beneath the thin coating resulted in the premature failure of the coating (see Figure 7, the W10 groups showed the early failure as fragmentation). Therefore, a balance between breathability and thickness is required to ensure the unimpeded permeation of the gaseous corrosive media with coating shelter lasting for a period of long enough time.

### 4.2. The Effect of Hydrophobization

During the salt spray test process, the fine water droplets would reach the sample surface and may accumulate into bigger droplets or form a continuous thin electrolyte film [65,66]. In more practical conditions, the condensate water and rainwater would influence the weathering steel. It may lead to the premature failure of the hydrophilic waterborne polyurethane (WPU) coating, presented as coating delamination, swelling, blistering and other phenomena [67,68,69].

When the accumulated liquid water stays on the surface of a WPU coating with high surface energy, the liquid water, together with dissolved corrosive ions, gradually penetrates into the coating through hydrogen-bonding adsorption along hydrophilic groups such as hydroxyl groups in the molecular chains [70]. The process has been reported to weaken the intermolecular forces of the coating, causing swelling and compromising the coating adhesion [71].

The undesired local penetration of liquid water would induce the non-uniformity of the rust layer and may result in “rust liquid” at a later stage. In other words, the concept seeks to block the ingress of liquid water, but the application of hydrophilic WPU would inevitably induce water damage on the coating [72], leading to the premature failure of the coating before the formation of a protective rust layer.

By incorporating hydrophobically modified hollow SiO_2_ nanoparticles, the low-surface-energy functional groups (-Si(CH_3_)_3_) together with the nanoscale size effect of the nanoparticles themselves, construct a rough structure on the WPU coating surface. This process significantly decreases the surface free energy of the coating and reduces the contact area with water droplets, altering the WPU coating from hydrophilic to hydrophobic [73]. The hydrophobic modification improves the wetting behavior of the coating surface, inhibiting the formation of a continuous thin electrolyte film or the accumulation of liquid water droplets. Thus, the actual contact area or the contact time of water droplets or a thin film can be reduced [74]. In addition, the self-cleaning property also largely avoids the influence of rust on the coating surface. Therefore, the negative effects of a thin electrolyte film or liquid water on the coating surface can be ruled out, ensuring the durability of the WPU-based coating. The maintenance of coating coverage was found to be improved after hydrophobization, especially at the early stage of rust formation, and the premature failure of the coating film was also mitigated (Figure 7, 7 days), implying an improved sheltering period for rust formation at an initial stage.

### 4.3. The Early Shelter of the Coating

As the protective rust layer gradually forms, the coating will undergo local damage due to rust expansion and eventually detach from the steel surface after the rust layer completely covers the substrate, replacing the coating [75]. Therefore, the concept of the breathable coating is to provide early shelter at the initial stage of rust formation, rather than offering protection throughout the service life, which does not obey the concept of self-protection for weathering steels.

Since it is impossible to directly and quickly form a compact and stable rust layer, the early failure of the coating would lead to insufficient time of shelter and the early exposure of an unstable and premature rust layer, so the initial stage (the most significant stage) would be disturbed. As shown in Figure 6, Figure 7 and Figure 8, the W10 (thickness 10 µm) coatings were prone to premature failure, resulting in early direct exposure of the rust layer to the salt spray environment and thus a loose rust layer, and chloride ingress could not be effectively inhibited (Figure 9).

In contrast, the W40 (thickness 40 µm) coating effectively delayed the coating failure and the direct exposure of the rust layer, offering a longer time of shelter, thereby enhancing the compactness and protective performance of the rust layer. The W80 (thickness 80 µm) coating did not experience severe failure or detachment even at the end of the 28-day salt spray. However, its rust layer formation process was very slow, and the metal surface was not completely covered at the end of the test (Figure 6). Furthermore, as observed in Figure 7, obvious cracks appeared on the W80_h-Si_ sample, with rust particles being pushed out from these cracks and distributed nearby, while there was still no obvious rust elsewhere. Although a remarkable area of coating was undamaged and the substrate was still covered by coating, this process actually exacerbated the non-uniformity of the rust layer and disfavored its protectiveness.

### 4.4. The Prospects of the Application

The breathable coating developed in this work employs waterborne polyurethane (WPU) and hollow SiO_2_ nanoparticles as the main raw materials, which are of low cost and contain no hazardous components, thereby avoiding the risk of environmental pollution. The coating film is based on a one-step spraying process that simplifies the application procedure and eliminates the influence of construction quality. The coating is able to provide effective shelter at the most important initial stage of rust layer formation, which helps fulfill the self-protection ability of weathering steels through a protective rust layer. In particular, the hydrophobic modification prevents the retention of liquid water on the steel surface and exhibits self-cleaning properties on the coating surface, largely mitigating the influence of typical factors that inhibit rust layer formation in the practical service environment, such as rainwater, pollutants and dust. As a novel rust layer stabilization technology for weathering steel, the breathable coating developed in this work can be widely applied in prospect for new constructions.

## 5. Conclusions

This study synthesized SiO_2_ nanoparticles with a mesoporous shell and hollow interior structure, followed by hydrophobic modification. The nanoparticles were used as fillers in waterborne polyurethane (WPU), and a hydrophobic and breathable coating film was fabricated to be applied for the rust stabilization of weathering steels. The main findings are summarized as follows.

(1) The coating facilitates the inward diffusion of gaseous corrosive media (H_2_O, O_2_ and Cl^−^ dissolved in moisture) while inhibiting the ingress of liquid water and dust to the coating/substrate interface, and a rust layer can form beneath the coating, which provides shelter for the initial stage of rust development against the disturbance of the external environment.

(2) The breathability of the coating is achieved by the effective interconnection of SiO_2_ nanoparticles across the coating film thickness, which provides a pathway for gaseous corrosive media. The breathability is inversely related to coating thickness, but shows no significant correlation with the hydrophobic modification.

(3) There exists a balance between the thickness (related to breathability) and effective sheltering period. The uniformity, the compactness, the stability, the inhibition of chloride ingress and corrosion resistance of the rust layer have been found to be optimized for the 40 μm thick hydrophobized coating film. A 10 μm thickness resulted in premature failure of the coating film, while an 80 μm thickness resulted in poor breathability, largely delayed rust development and the non-uniformity of the rust layer.

(4) The hydrophobization of the nanoparticles helps achieve a hydrophobic coating surface, which enhances the water resistance of the naturally hydrophilic WPU and mitigates the premature failure of the coating film.

(5) The breathable coating developed in this study demonstrates prospects owing to its maneuverability, environmental friendliness and low cost, which provides a new technical route for rust stabilization of weathering steels.

## Figures and Tables

**Table 1:** A summary of different types of polyurethane-based coatings and their properties.

Polyurethane Coating Types	Solvent Type	Advantages	Defects	Primary Fields of Application
Solvent-based polyurethane (PU)	Organic solvent	Excellent mechanical strength, along with abrasion, chemical and weather resistance [34].	High VOC emissions; flammable organic solvents; deterioration in mechanical properties under UV light [35,36].	Harsh environments and mechanical stress, widely used in industries, oil and gas facilities, architectural finishes, etc. [28].
Waterborne polyurethane (WPU)	Water	Less VOCs, low temperature flexibility, pH stability, solvent resistance, weather resistance and mechanical properties [37].	Residual trace organic solvents; prolonged drying time, insufficient initial tack, and poor water resistance [38,39].	Widely applied in anticorrosion coatings, medical products, textiles, leather, etc. [39].
Solvent-free polyurethane (SFPU)	No organic solvent added	No VOC, favorable storage stability, high flexibility and mechanical strength, along with self-cleaning, anticorrosion and antifouling properties [38,40,41].	High curing rate deteriorates hygienic performance, such as coating breathability and moisture permeability; long synthesis time and high energy consumption [42,43].	Suitable for sustainable textile coatings; extensively used in industrial protection and daily life scenarios [38,41].

**Table 2:** Chemical composition of Q420qDNH weathering steel.

Element	C	Si	Mn	V	Cr	Ni	Cu	Fe
Mass fraction %	0.09	0.31	1.24	0.008	0.42	0.36	0.35	Bal.

**Table 3:** Results of the fitting analysis after the 28-day salt spray test.

Sample	R_s_ (Ω·cm^2^)	Y_r_ × 10^−2^ (Ω^−1^·cm^−2^·s^n^)	n_r_	R_r_ (Ω·cm^2^)	Y_dl_ × 10^−2^ (Ω^−1^·cm^−2^·s^n^)	n_dl_	R_ct_ (Ω·cm^2^)	Χ^2^ × 10^−4^
Bare steel	22.7	1.948	0.689	30.0 ± 3.0	4.088	0.640	98.9 ± 6.1	3.663
W10_Si_	17.7	1.676	0.691	32.0 ± 3.1	2.946	0.678	108.7 ± 11.1	1.024
W10_h-Si_	13.8	1.233	0.742	39.3 ± 0.5	2.202	0.655	121.8 ± 4.5	2.725
W40_Si_	26.0	0.936	0.820	55.7 ± 1.2	0.810	0.748	138.4 ± 2.5	5.254
W40_h-Si_	27.6	0.929	0.890	65.1 ± 2.0	0.471	0.816	149.8 ± 3.9	3.511
W80_Si_	33.7	1.432	0.726	44.3 ± 2.7	2.017	0.707	129.6 ± 3.9	2.090
W80_h-Si_	32.0	1.229	0.799	48.8 ± 0.2	1.616	0.780	136.3 ± 4.6	4.193

**Table 4:** Corrosion potential and corrosion current density values of different samples after the 28-day salt spray test.

Sample	E_corr_ (V/SCE)	I_corr_ (A/cm^2^)
Bare steel	−0.571	7.18 × 10^−4^
W10_Si_	−0.566	4.24 × 10^−4^
W10_h-Si_	−0.562	3.62 × 10^−4^
W40_Si_	−0.539	1.38 × 10^−4^
W40_h-Si_	−0.536	1.22 × 10^−4^
W80_Si_	−0.559	3.49 × 10^−4^
W80_h-Si_	−0.541	2.86 × 10^−4^

## Data Availability

The raw data supporting the conclusions of this article will be made available by the authors on request.
